# Case Report of Surgical Management of Duodenal Perforation Secondary to Stent in a Patient With Gastric Outlet Obstruction Due to a Rare Neuroendocrine Adenocarcinoma of Duodenum

**DOI:** 10.7759/cureus.19562

**Published:** 2021-11-14

**Authors:** Kyle Drinnon, Yana Puckett

**Affiliations:** 1 Surgery, Texas Tech University Health Sciences Center, Lubbock, USA; 2 Surgery, West Virginia University School of Medicine, Charleston, USA

**Keywords:** duodenal perforation, well differentiated neuroendocrine tumor, duodenal stent, intestinal perforation, whipple procedure

## Abstract

Neuroendocrine adenocarcinomas of the duodenum comprise a rare subset of neuroendocrine tumors and commonly present with symptoms of gastric outlet obstruction (GOO). Most of the time, patients are recommended a GI bypass in the setting of metastatic disease. In a small subset of patients who prefer a non-operative approach or are poor surgical candidates, duodenal stenting can often accomplish similar results as surgery. However, duodenal stenting is associated with numerous complications, including duodenal stent migration and, less commonly, duodenal perforation. We present a case where duodenal stenting resulted in a perforation of the second portion of the duodenum that ultimately required a definitive pancreaticoduodenectomy.

## Introduction

Patients with gastroduodenal malignancies may experience a decline in quality of life in part, due to difficulty eating, vomiting, and subsequent weight loss accompanying those symptoms. Duodenal stenting with self-expandable metal stents (SEMS) can provide an alternative to surgical bypass procedures in patients that this may be contraindicated in or in those who prefer to undergo a less invasive procedure [1]. However, the placements of these stents are not without complications. Stent perforation, as seen in this case report, occurs in up to 5% of patients, leading to the patient’s death in 1% of these procedures {2,3]. Perforations that occur during the placement of the stent can occur due to the guidewire or after balloon dilation. However, in most cases, the perforation is recognized after the procedure is done. In most cases, when a duodenal stent perforation occurs, patients complain of severe abdominal pain, and a CT scan of the abdomen shows the presence of free air [2]. We present a case where a duodenal SEMS resulted in a perforation of the second portion of the duodenum that ultimately required a definitive pancreaticoduodenectomy.

## Case presentation

The patient is an 82-year-old male that presented with metastatic, mixed adenocarcinoma/neuroendocrine duodenal cancer. A pancreaticoduodenectomy surgery (Whipple) for definitive treatment was offered to the patient. The patient was reluctant to undergo major surgery and opted for systemic treatment instead. He presented to the ER two months after starting chemotherapy with symptoms of gastric outlet obstruction (GOO) (vomiting, belching, and weight loss). An attempt was made to manage the GOO non-operatively per the patient’s wishes with duodenal stenting.

**Figure 1:**
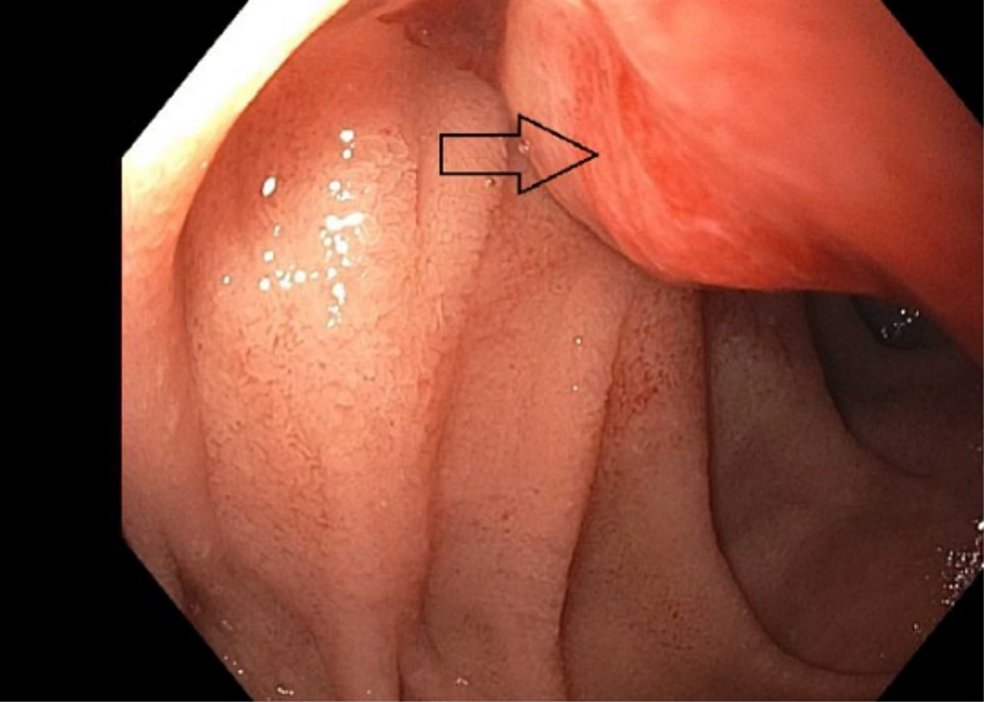
A partial obstruction, mildly ulcerated mass was seen in the second portion of the duodenum of the patient on esophagogastroduodenoscopy.

A self-expanding, 22 mm x 9 cm fully covered, metal stent was placed into the second portion of the duodenum where a large, partially obstructing, ulcerated mass was visualized endoscopically (Figure 1).

**Figure 2:**
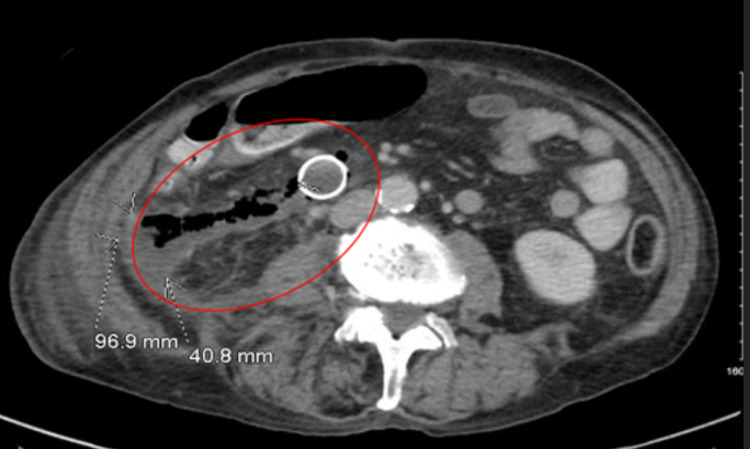
Cross-sectional imaging of the abdomen showing expanding stent perforation, with evidence of free air as well as food particles inside of the abdomen creating a large 96.9 mm by 40.8 mm fluid collection.

Several hours after the procedure, the patient complained of severe abdominal pain. A CT scan of the abdomen revealed that the stent placement resulted in a perforation of the duodenum with extension into the retroperitoneum (Figure 2).

Emergency pancreaticoduodenectomy versus damage control surgery were the two surgical options offered to the patient. The patient opted for damage control surgery. He was taken emergently for an exploratory laparotomy, washout of the abdomen, wide drainage, duodenostomy tube placement, and pyloric exclusion.

His postoperative course was uneventful, and he recovered quickly. After four months of healing, the patient underwent a definitive pancreaticoduodenectomy procedure. Final pathology revealed a mixed adeno-neuroendocrine carcinoma, arising from the duodenum/peri-ampullary area and invading into the pancreas and duodenal serosa (pT3, pN1, cM1) with 3 of 16 lymph nodes positive for metastatic carcinoma and all margins negative. He went on to receive adjuvant systemic treatment and continues to do well postoperatively.

## Discussion

We present a case of a patient with a metastatic duodenal neuroendocrine tumor who opted to decline definitive treatment with a pancreaticoduodenectomy initially. The patient developed a GOO treated with duodenal stent placement. The patient incurred a duodenal perforation secondary to stent placement requiring a damage-control surgery. After recovery, the patient was then treated with a Whipple procedure four months later. This case report highlights a difficult clinical situation in a patient's declining standard of care treatment. This case report highlights the importance of being able to deal with complications in a palliative care setting and highlights a different option of treatment. 

Patients with gastroduodenal malignancies tend to have worse quality of life compared to malignancies in other parts of the body in part, due to difficulty eating, vomiting, and subsequent weight loss accompanying those symptoms. Duodenal stenting with SEMS can provide a non-surgical alternative to GI bypass procedures in patients who may refuse surgery or in those for whom comorbidities preclude a surgical procedure [1]. However, endoscopic stent placements are not without complications.

GI perforation due to stent placement can occur in up to 5% of patients, leading to the patient’s death in 1% of these procedures [2,3]. Perforations that occur during the placement of the stent are usually due to guidewire insertion [4]. In most cases, perforations are recognized after placement when patients complain of severe abdominal pain and a CT scan of the abdomen shows the presence of free air [4] as was seen in our patient.

Management of GI perforations from stent placement is commonly managed with an endoscopic repair that can entail stent removal and clip placement to seal the perforation or endoscopic placement of a covered stent if the perforation is small and the patient has no evidence of peritonitis on clinical exam [3,4]. In our case, the patient had evidence of peritonitis on exam and was deteriorating clinically. As such, an endoscopic repair was not a safe option.

Recent research suggests that surgery may be a more favorable treatment plan when patients have a life expectancy greater than two months, while patients with advanced or metastatic disease and patients experiencing malnutrition and biliary obstruction may benefit more from stent placement [4]. Even with these recommendations, SEMS is still the treatment of choice in some institutions due to its less invasive nature and small complication rate [4].

The treatment for duodenal stent perforation consists of abdominal washout, removal of the sent, and resection of the perforation. Most often, this involves pyloric exclusion, wide drainage, and a jejunostomy tube away from the perforation to accommodate enteral nutrition. After that, a period of healing and inflammation takes place and definitive surgery should not take place for 4-6 months [5].

The performance of an emergency pancreaticoduodenectomy in the setting of frank GI perforation carries with it a high risk of morbidity and mortality for the patient. However, in a healthy young patient, this may be an option [5]. In our case scenario, the patient was resistant to having surgery for his neuroendocrine cancer in the first place. As such, a damage-control operation was decided to be the best option for him. 

The definitive treatment of duodenal stent perforation is segmental resection and anastomosis if in the third or fourth portion of the duodenum. If the perforation occurred at the second portion of the duodenum, then the definitive operation is a pancreaticoduodenectomy or otherwise known as the Whipple procedure [6].

## Conclusions

Duodenal stenting for malignant GOO is commonplace. However, one should be familiar with the management of duodenal stent placement complications such as migration and less commonly, perforation of the duodenum.
